# A Critical Role of Autophagy in Regulating Microglia Polarization in Neurodegeneration

**DOI:** 10.3389/fnagi.2018.00378

**Published:** 2018-11-20

**Authors:** Meng-meng Jin, Fen Wang, Di Qi, Wen-wen Liu, Chao Gu, Cheng-Jie Mao, Ya-Ping Yang, Zhong Zhao, Li-Fang Hu, Chun-Feng Liu

**Affiliations:** ^1^Department of Neurology, Suzhou Clinical Research Center of Neurological Disease, Suzhou Municipal Hospital of Nanjing Medical University, Suzhou, China; ^2^Department of Neurology, The Second Affiliated Hospital of Soochow University, Suzhou, China; ^3^Department of Neurology, University Hospital Carl Gustav Carus, Technical University of Dresden, Dresden, Germany; ^4^Jiangsu Key Laboratory of Neuropsychiatric Diseases, Institute of Neuroscience, Soochow University, Suzhou, China; ^5^Department of Pharmacology, College of Pharmaceutical Sciences, Soochow University, Suzhou, China

**Keywords:** microglia polarization, TNF-α, autophagy, inflammation, neurodegeneration

## Abstract

Neuroinflammation and autophagy dysfunction are closely related to the development of neurodegeneration such as Parkinson’s disease (PD). However, the role of autophagy in microglia polarization and neuroinflammation is poorly understood. TNF-α, which is highly toxic to dopaminergic neurons, is implicated as a major mediator of neuroinflammation in PD. In this study, we found that TNF-α resulted in an impairment of autophagic flux in microglia. Concomitantly, an increase of M1 marker (iNOS/NO, IL-1β, and IL-6) expression and reduction of M2 marker (Arginase1, Ym1/2, and IL-10) were observed in TNF-α challenged microglia. Upregulation of autophagy via serum deprivation or pharmacologic activators (rapamycin and resveratrol) promoted microglia polarization toward M2 phenotype, as evidenced by suppressed M1 and elevated M2 gene expression, while inhibition of autophagy with 3-MA or Atg5 siRNA consistently aggravated the M1 polarization induced by TNF-α. Moreover, Atg5 knockdown alone was sufficient to trigger microglia activation toward M1 status. More important, TNF-α stimulated microglia conditioned medium caused neurotoxicity when added to neuronal cells. The neurotoxicity was further aggravated when Atg5 knockdown in BV2 cells but alleviated when microglia pretreatment with rapamycin. Activation of AKT/mTOR signaling may contribute to the changes of autophagy and inflammation as the AKT specific inhibitor perifosine prevented the increase of LC3II (an autophagic marker) in TNF-α stimulated microglia. Taking together, our results demonstrate that TNF-α inhibits autophagy in microglia through AKT/mTOR signaling pathway, and autophagy enhancement can promote microglia polarization toward M2 phenotype and inflammation resolution.

## Introduction

Parkinson’s disease (PD) is a leading neurodegenerative movement disorder, caused by the demise of dopaminergic neurons in the substantia nigra (SN). Although the molecular mechanisms that underlie dopaminergic neuron losses are still unclear, it is increasingly recognized that microglia-mediated neuroinflammation serves as a pathogenic factor in PD (Glass et al., 2010). Accumulation of proinflammatory cytokines, such as tumor necrosis factor-α (TNF-α), interleukin-1β (IL-1β), and interleukin-6 (IL-6) was reported in the brain, cerebrospinal fluid and serum of patients with PD, and animal models as well (Hunot and Hirsch, 2003). The polymorphism of human TNF-α encoding gene increases the risk of developing PD. The plasma TNF-α level correlates with some of the non-motor symptoms in PD including cognition decline, sleep disruption, and depression (Menza et al., 2010). At the molecular level, our previous study identified the autophagy deficits in TNF-α treated dopaminergic cells (Wang et al., 2015). All these suggest that TNF-α is a critical mediator in PD pathogenesis (Mccoy et al., 2011).

The microglia density is remarkably higher in the SN compared to other regions (Kim et al., 2000). Microglia exerts neurotoxic and neuroprotective effects, depending on their diverse functional phenotypes in response to specific stimuli (Orihuela et al., 2016). “Classic M1 polarization” of microglia is characterized by the production of pro-inflammatory mediators. In contrast, alternatively polarized M2 microglia limit inflammation and are typically characterized by the yield of anti-inflammatory factors (Kigerl et al., 2009; Hu et al., 2012). The enhanced microglial M1 activation contributes to neurodegeneration, such as PD and Alzheimer’s disease (Wyss-Coray and Rogers, 2012; Vivekanantham et al., 2015). However, the molecular pathway that drives the microglia phenotypic changes during neurodegeneration remains unclear.

Autophagy is a catabolic mechanism which removes unnecessary or dysfunctional proteins and damaged organelles via the lysosome machinery (Mizushima, 2007). Autophagy dysregulation, along with neuroinflammation, is implicated in PD pathogenesis (Plaza-Zabala et al., 2017). The interaction between autophagy and inflammation is very complicated and controversial. For instance, in lipopolysaccharide (LPS)-challenged macrophages, Atg16L1 (an autophagy-related protein) deficiency enhanced IL-1β production (Saitoh et al., 2008). Nevertheless, there was also study reported that rapamycin, an autophagy activator, can inhibit M2 macrophage polarization (Wang et al., 2017). However, it remains unclear how autophagy regulates the shifting between M1 and M2 phenotypes in microglia, the macrophage counterpart in the brain. Therefore, our present study was to explore if autophagy has any impact on the microglial polarization upon TNF-α exposure, and the molecular mechanisms involved.

## Materials and Methods

### Reagents and Antibodies

The chemicals such as 3-methyladenine (3-MA), bafilomycin A1 (BafA1), resveratrol, rapamycin were purchased from Sigma-Aldrich (St. Louis, MO, United States). TNF-α was purchased from PEPROTECH (Rocky Hill, NJ, United States). The Perifosine was purchased from Selleck Chemicals (Houston, TX, United States). The sources for primary antibodies were listed as follows: anti-β-actin, anti-p62, anti-lysosome-associated membrane protein 1 (LAMP1) and anti-β-tubulin from Sigma-Aldrich (St. Louis, MO, United States); anti-LC3, anti-histone 2B and anti-LAMP2 (Abcam, Cambridge, United Kingdom), anti-p-mammalian target of rapamycin (mTOR) [Cell Signaling Technology (CST), 5536s], anti-mTOR (CST, 2983s), anti-p-AKT (CST, 4060), anti-AKT (CST, 4691) and anti-cleaved caspase-3 (CST, 9664); anti-transcription factor EB (TFEB) (Proteintech, Chicago, IL, United States), and anti-GAPDH (Millipore, Billerica, MA, United States). All the reagents for cell culture were bought from Gibco (Grand Island, United States).

### Cell Culture and Treatment

Murine BV2 microglial cells were cultured in Dulbecco modified Eagle’s medium (DMEM) supplemented with 10% fetal bovine serum (FBS) and 1% penicillin/streptomycin in an incubator with 95% air atmosphere/5% CO_2_ at 37°C. MES23.5 cells were cultured in DMEM/F12 with Sato’s components containing 5% heat-inactivated FBS with 1% penicillin/streptomycin in the incubator. Cells were regularly subcultured three times a week and seeded into 35 mm dishes or 24-well plates prior to experimentation.

Primary microglia culture was prepared from 1 to 2-day-old neonatal C57BL/6J mice. Briefly, the cortex was dissected, minced and dissociated in 0.125% Trypsin for 4 min at 37°C. Trypsin was then neutralized with complete media (DMEM/F12 supplemented with 15% heat-inactivated FBS) and strained through a 200 μm mesh filter. After brief centrifugation, cells were harvested and plated in T75 cell culture flasks. The culture medium was replaced every 3 days. Once the lower layer reached confluence (about 10 days after culture), microglia cells were harvested by mechanical agitation at 180 rpm for 60–90 min and subsequently plated in DMEM/F12 supplemented with 10% FBS at a desired density for further experimentation.

### Quantitative PCR

Total RNA was extracted using Trizol reagent and reverse transcribed using cDNA synthesis kit (Fermentas, Vilnius, Lithuania). Quantitative PCR (qPCR) was performed as we previously elaborated (Zhou et al., 2014). The primers used were listed in Table 1. 18s RNA was used as an internal control. The results were normalized and expressed as ratios of the target gene over 18s mRNA level.

**Table 1:** The Primers Used for Quantitative PCR.

Primer	Forward (5′ to 3′)	Reverse (5′ to 3′)
p62 iNOS IL-1β	CAGGCGCACTACCGCGATGA CAGGAGGAGAGAGATCCGATTTA TGGAAAAGCGGTTTGTCTTC	TCGCACACGCTGCACAGGTC GCATTAGCATGGAAGCAAAGA TACCAGTTGGGGAACTCTGC
Arginase1	GAACACGGCAGTGGCTTTAAC	TGCTTAGCTCTGTCTGCTTTGC
Ym1/2	CAGGGTAATGAGTGGGTTGG	CACGGCACCTCCTAAATTGT
18 s	TCAACACGGGAAACCTCAC	CGCTCCACCAACTAAGAAC

### Nitrite Quantification

The nitric oxide (NO) production in the culture supernatant was detected using a kit from Beyotime Biotechnology (Shanghai, China). Briefly, 50 μl culture supernatant was collected after treatment and transferred into a 96-well plate. Then, 50 μl Griess reagent was added and incubated at room temperature for 10 min. The absorbance was measured by a Microplate Reader (Tecan M200, Grodig) with a wavelength at 540 nm. The NO level was calculated with a standard curve plotted by sodium nitrite.

### Cytokine Assay

The content of the cytokines IL-6 and IL-10 in the culture supernatant was detected using the ELISA kits from BOSTER (Wuhan, China), according to the manufacturer’s instructions.

### Western Blot Analysis

Whole cell lysates were washed prepared by washing cells with phosphate-buffered saline (PBS) and lysed in ice-cold lysis buffer (150 mM NaCl, 25 mM Tris, 5 mM EDTA, 1% Nonidet P-40, pH 7.5) as previously described (Du et al., 2014). Lysates were separated by 10% sodium dodecyl sulfate-polyacrylamide gels and transferred onto nitrocellulose membranes. Next, membranes were blocked in 5% (w/v) non-fat dry milk powder in 0.1% Tris-buffered saline/Tween 20 (TBST) for 1 h and then incubated with the primary antibodies at optimized dilutions at 4°C overnight. After that, membranes were washed with TBST and incubated with HRP-conjugated secondary antibodies for another 1 h. Blots were finally visualized with chemiluminescence (Thermo Company, West Chester, PA, United States) and band densities were analyzed with ImageJ software (National Institute of Health, United States).

### RFP-GFP-Tandem Fluorescent-Tagged LC3 (tf-LC3) and Atg5 siRNA Transfection

For transfection, 0.8 μg RFP-GFP-LC3 cDNA (Addgene, United States) were transfected into BV2 cells using 2 μl Lipofectamine 2000 (Invitrogen, Eugene, OR, United States). To monitor autophagic flux, cells were exposed to TNF-α or vehicle with or without the lysosome inhibitor BafA1 at 24 h post-transfection with tf-LC3 plasmids. The yellow and red LC3 puncta were manually counted, and at least 30 cells per group were randomly selected for counting. The small-interfering RNAs (siRNA) targeting mouse Atg5 (5′GACGUUGGUAACUGACAAATT3′ and 5′UUUGUCAGUUACCAACGUCTT3′) and scrambled siRNA duplexes were synthesized by GenePharma (Shanghai, China), and were transfected using Lipofectamine RNAiMAX (Thermo Company). The knockdown efficiency was determined by western blotting at 24 h post-transfection.

### Subcellular Fractionation

Cells were lysed in the fractionation buffer including 3 mM CaCl_2_, 2 mM MgAc, 320 mM sucrose, 0.1 mM EDTA, 1 mM DTT, 0.5 mM phenylmethylsulfonyl fluoride (PMSF), and 0.5% NP-40, kept on ice for 20 min and then centrifuged at 600 *g* 4°C for 15 min. The supernatants were collected as the cytosolic fraction. The pellets were washed with fractionation buffer without NP-40 twice, lysed with the nuclear lysis buffer comprising 280 mM KCl, 0.2 mM EDTA, 1 mM DTT, 0.5 mM PMSF, 20 mM Hepes (pH 7.9), 25% glycerol, 1.5 mM MgCl_2_, and 0.3% NP-40 and then centrifuged. The resulting supernatant was then used for the nuclear fraction.

### Conditioned Medium-Induced Neuronal Cell Death Assay

BV2 cells were transfected with Atg5 siRNA or control siRNA for 48 h as mentioned above. For rapamycin group, cells were pretreated with 0.2 μg/ml rapamycin for 0.5 h. Cells were then treated with TNF-α for 24 h, and replaced by culture in fresh medium for another 24 h to produce the microglia conditioned medium (CM). After that, the microglia CM was transferred into MES23.5 cells and cultured for 24 h. Thereafter, cells were incubated with Hochest 33342 (Sigma) and propidium iodide (PI, Beyotime, Shanghai, China) for 5 min, and then washed with PBS. Images were taken using an inverted IX71 microscope system (Olympus, Tokyo, Japan). The hochest and PI stained cells were counted manually, and at least 500 cells per group were counted.

### Statistical Analysis

All results are presented as mean ± SEM, obtained from a minimum of 3 independent experiments unless otherwise stated. Statistical significance was analyzed using Student *t*-test for two-group comparison or one-way analysis of variance (ANOVA) followed by Tukey *post hoc* analysis using the GraphPad Prism. The significance level was set at *P* < 0.05.

## Results

### TNF-α Enhances M1 but Reduces M2 Markers in Microglia

**FIGURE 1:**
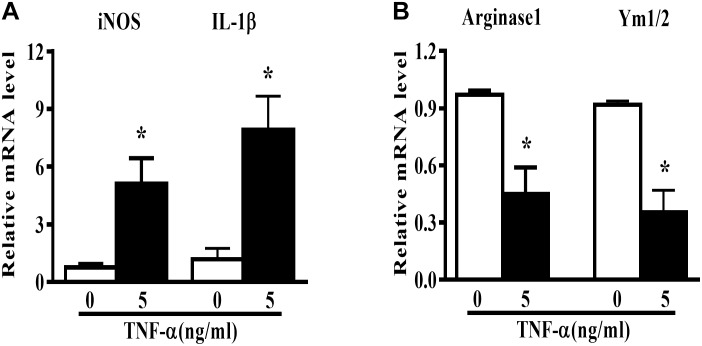
TNF-α enhances M1 but reduces M2 markers in mouse primary microglia. Quantitative PCR (qPCR) analysis of M1 genes (iNOS, IL-1β, **A**) and M2 genes (Arginase1 and Ym1/2, **B**) mRNA levels in mouse primary microglia at 6 h after 5 ng/ml TNF-α stimulation, *n* = 4. Graphs show relative mRNA levels after normalization with those of housekeeping gene 18 s correspondingly. Results are expressed as mean ± SEM of four independent experiments. Student *t*-test. ^∗^*P* < 0.05 vs. untreated controls.

Microglia polarization is commonly characterized by the expression of signature genes that are associated with M1 or M2 phenotype (Mantovani et al., 2013). Neuroinflammation is featured by an alteration of microglia polarization toward M1 status. In this study, we observed that 5 ng/ml TNF-α stimulation not only induced an elevation in M1 genes (iNOS and IL-1β) expression (Figure 1A), but also caused a reduction in the M2 signature genes level (Arginase1 and Ym1/2, Figure 1B) in mouse primary microglia, indicating a shift toward M1 phenotype following TNF-α stimulation.

### Autophagy Activation Enhances M2 but Reduces M1 Markers in Microglia

**FIGURE 2:**
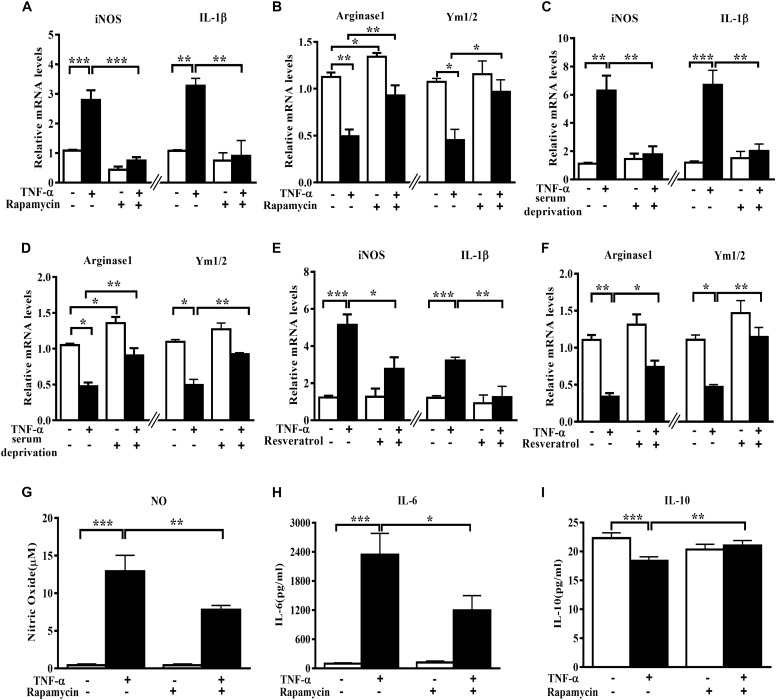
Autophagy stimulation enhances M2 but reduces M1 markers in primary microglia. **(A–F)** qPCR analysis of M1 and M2 genes mRNA levels in TNF-α-treated primary microglia with or without autophagy stimulation by different methods. **(A,B)** 0.2 μg/ml rapamycin pretreatment. **(C,D)** serum deprivation. **(E,F)** autophagy stimulation by 50 μM resveratrol pretreatment. Graphs show relative mRNA levels after normalization with those of corresponding housekeeping gene 18 s, *n* = 4. **(G–I)** Cytokines including NO **(G)**, IL-6 **(H)** and IL-10 levels **(I)**, measured by Griess reagent and ELISA, in cell-free culture supernatant from TNF-α-challenged primary microglia with or without rapamycin pretreatment. *N* = 4. All of the results are expressed as mean ± SEM, one-way ANOVA followed by Tukey analysis. ^∗^*P* < 0.05, ^∗∗^*P* < 0.01, and ^∗∗∗^*P* < 0.001 vs. controls. NS, not significant.

In order to assess the role of autophagy in microglia polarization, we induced autophagy activation in primary microglia using serum deprivation or two structure-unrelated autophagy inducers (rapamycin and resveratrol). Although none of the three tested autophagy inducers produced any obvious effect on the basal level of M1 genes, they increased the Arginase1 mRNA levels under normal conditions. A mild but not significant elevation of Ym1/2 mRNA was also observed. Importantly, both serum deprivation and pharmacologic autophagy inducers (rapamycin and resveratrol) potently suppressed the increase of M1 (iNOS and IL-1β) mRNA level and alleviated the decline of M2 gene expression (Arginase1 and Ym1/2) in TNF-α-stimulated primary microglia (Figures 2A–F). Measurement of the cytokines level in the culture supernatant displayed that the autophagy activator rapamycin reduced the NO (Figure 2G) and IL-6 (Figure 2H) production but enhanced IL-10 yield (Figure 2I) in TNF-α-stimulated primary microglia. These data imply that autophagy activation is able to promote microglia polarization toward M2 phenotype under both basal and inflamed status.

### Autophagy Inhibition Enhances M1 but Reduces M2 Markers in Microglia

**FIGURE 3:**
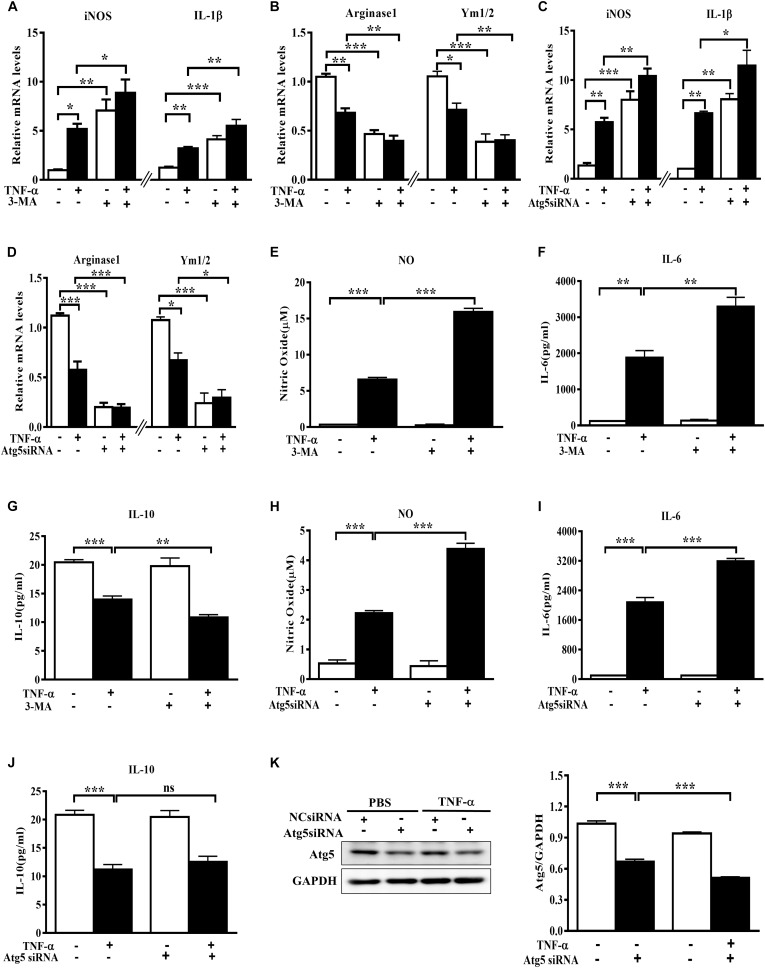
Autophagy inhibition enhances M1 but reduces M2 markers in microglia. **(A–D)** qPCR measurement of M1 and M2 genes mRNA levels in TNF-α-treated cells with or without autophagy inhibition by 10 mM 3-MA (**A,B**, primary microglia) or Atg5 knockdown (**C,D**, BV2 microglial cell line). Atg5 siRNA or scrambled siRNA were transfected into BV2 cells by Lipofectamine RNAiMAX, followed by 5 ng/ml TNF-α treatment at 24 h later. mRNA levels were analyzed at 6 h after TNF-α addition. Graphs show relative mRNA levels after normalization with those of corresponding housekeeping gene 18 s. *N* = 3–6. **(E–J)** NO, IL-6 and IL-10 levels in the culture supernatants from TNF-α-treated microglia with 3-MA or Atg5 siRNA as described above, *n* = 4. **(K)** Western blot study of Atg5 expression in Atg5 siRNA-transfected BV2 cells following TNF-α treatment, *n* = 3. Housekeeping protein GAPDH was used as loading control for semi-quantitative densitometry. ^∗^*P* < 0.05, ^∗∗^*P* < 0.01, and ^∗∗∗^*P* < 0.001 vs. controls, one-way ANOVA followed by Tukey analysis.

To evaluate the role of autophagy inhibition in microglia phenotype transition, we used both the autophagy inhibitor 3-MA and the siRNA against autophagy-related protein Atg5 and observed the changes of polarization markers in TNF-α-challenged microglia cells. The autophagy inhibitor 3-MA enhanced the basal and TNF-α-induced iNOS and IL-1β transcription (Figure 3A). Meanwhile, it downregulated the basal level of Arginase1 and Ym1/2 and caused a further decline of these two M2 markers in TNF-α-exposed primary microglia (Figure 3B). In line with this, Atg5 knockdown in BV2 cells was also found to increase the basal level of M1 gene expression (iNOS, IL-1β) and reduce the M2 gene expression (Arginase1, Ym1/2) (Figures 3C–D), and reinforced the alterations of the polarization markers in TNF-α-stimulated cells. These observations were confirmed by cytokines assay. As shown in Figures 3E–J, autophagy inhibition with 3-MA or Atg5 siRNA consistently caused a further increase of NO and IL-6 generation and a moderate decrease of IL-10 in the supernatant of TNF-α-treated microglia. Western blot study revealed the successful knockdown of Atg5 by small RNA interference in TNF-α-treated BV2 cells (Figure 3K). These data indicate that autophagy not only negatively regulates microglia activation under basal conditions but also affects microglia polarization in response to inflammatory stimulation.

### TNF-α Disrupts Microglia Autophagic Flux

**FIGURE 4:**
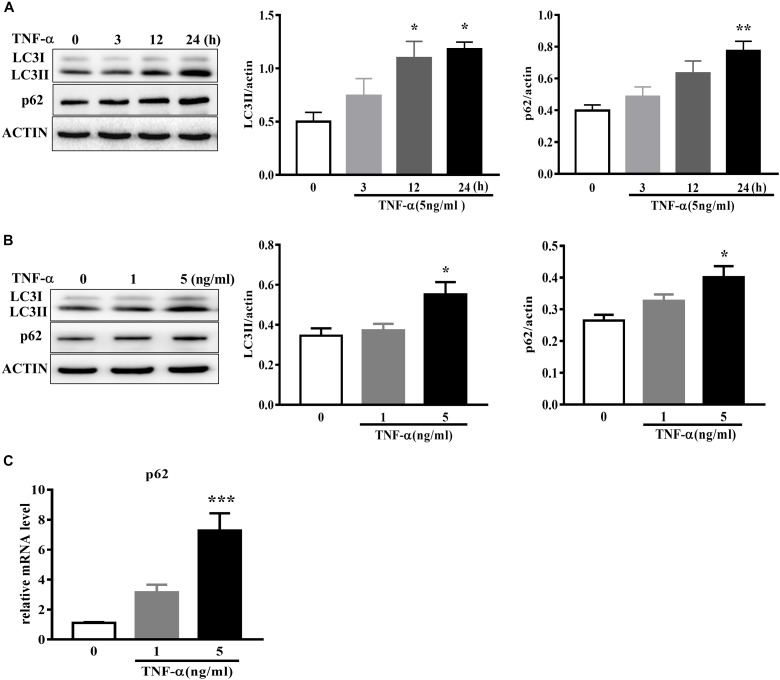
TNF-α disrupts autophagy in microglia. **(A,B)** Western blot analysis of the time- and dose-dependent effects of TNF-α on the autophagy markers LC3II and p62 protein levels in primary microglia. Actin served ad as loading controls, *n* = 4. **(C)** qPCR measurement of p62 mRNA expression in microglia following TNF-α treatment for 6 h. Relative p62 mRNA level was obtained by normalizing to that of housekeeping 18 s, *n* = 4. ^∗^*P* < 0.05, ^∗∗^*P* < 0.01, and ^∗∗∗^*P* < 0.001 vs. controls, one-way ANOVA followed by Tukey analysis.

To examine the effect of TNF-α on microglia autophagy, we study several autophagy-related markers including LC3 and p62 (also named sequestosome1, SQSTM1) protein expressions. Western blotting analysis showed that TNF-α treatment resulted in an increase of microglia LC3II and p62 in a time- and dose-dependent manner (Figures 4A–B). As several studies previously reported the p62 transcription by inflammatory stimuli (Fujita et al., 2011), here we also examined p62 mRNA level. qPCR analysis showed a substantial increase of p62 transcription in response to TNF-α stimulation (Figure 4C).

**FIGURE 5:**
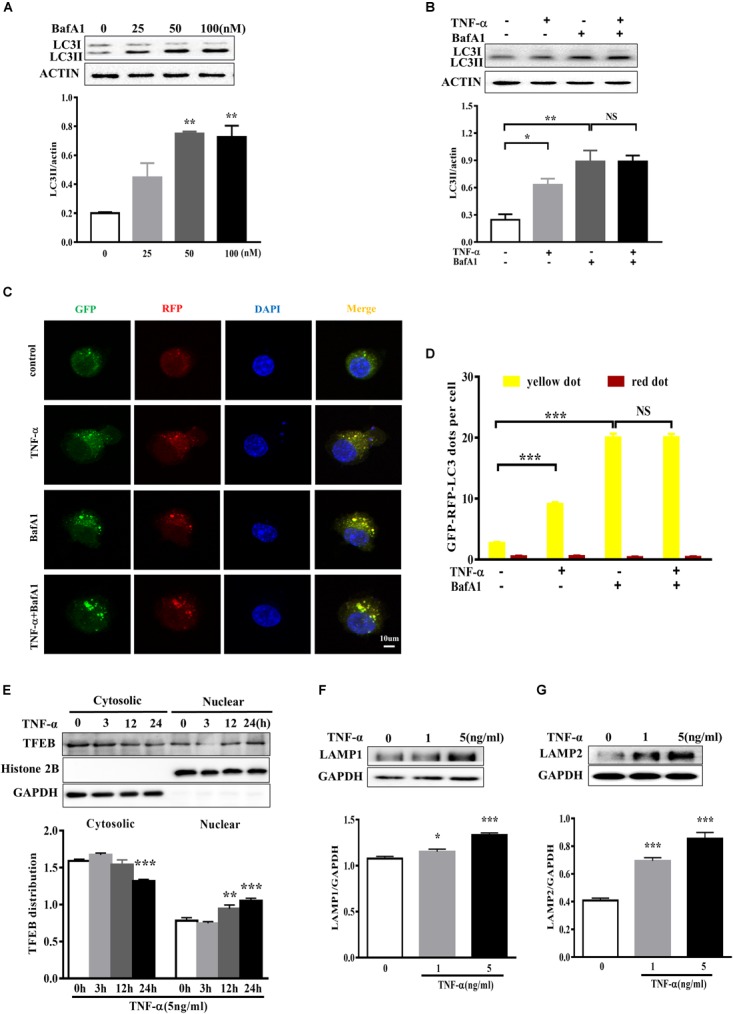
TNF-α disrupted the autophagic flux in microglia. **(A)** Dose-dependent effect of the lysosome inhibitor BafA1 treatment for 2 h on LC3II accumulation in BV2 cells, as evaluated by western blotting. *N* = 3. **(B)** Effect of TNF-α on LC3II levels during lysosome inhibition. BV2 cells were treated with 5 ng/ml TNF-α for 24 h, and then added with BafA1(50 nM) for 2 h before subjected to western blotting. Actin served as loading controls in panels **A,B**. *N* = 4. **(C,D)** Autophagic assay in microglia treated with TNF-α, BafA1, or in combination. BV2 cells were transfected with RFP-GFP-tandem fluorescent LC3 cDNA for 24 h before treatment. Confocal microscope pictures showing yellow (GFP and RFP overlap) and red LC3 puncta formation in different groups. Scale bar, 10 μm. LC3 dots were visualized and quantified from at least 30 cells per group. **(E)** Effect of TNF-α on lysosomal biogenesis. BV2 cells were treated with 5 ng/ml TNF-α for 3, 12, or 24 h. The cytosolic and nuclear fractions were subjected to western blotting analysis of TFEB, with GAPDH and Histone 2B as the cytosolic and nuclear loading controls, respectively. **(F,G)** Effect of TNF-α on lysosomal protein LAMP1 **(F)** and LAMP2 **(G)** levels in BV2 cells. *N* = 3. ^∗^*P* < 0.05, ^∗∗^*P* < 0.01, and ^∗∗∗^*P* < 0.001 vs. controls. NS, not significant. One way ANOVA followed by Tukey analysis.

To determine whether the LC3II increase resulted from autophagy induction or lysosomal degradation impairment, we studied the effect of TNF-α on LC3II in the presence of BafA1, a vacuolar H^+^-ATPase inhibitor that blocks autolysosome degradation. As shown in Figure 5A, showed that 50 nM BafA1 was sufficient to fully block of lysosomal degradation as 100 nM BafA1 did not result in a further increase of LC3II level in BV2 cells. Therefore, 50 nM BafA1 was used in the following study for lysosomal inhibition. TNF-α failed to further enhance the LC3II protein level in the presence of BafA1 (Figure 5B), indicating that the TNF-α-induced LC3II increase may result from lysosome degradation impairment. This is consistent with the tf-LC3 assay which allows us to monitor the autophagic flux (Mizushima et al., 2010). As shown in Figure 5C, tf-LC3 transfected BV2 microglia had some basal level of autophagy, as exhibited by a few green/red dots staining in control. Similar to the impact of BafA1, TNF-α caused an obvious elevation in the number of yellow dots, with almost no detectable red dots in cells. TNF-α plus BafA1 co-treatment did not show any further impact on the yellow/red dots when compared to BafA1 treatment alone (Figures 5C,D). These data suggest the disruption of autophagic flux in TNF-α challenged microglia.

Anti-transcription factor EB served as a master regulator of the autophagy-lysosome system which coordinates autophagy and lysosomal biogenesis (Sardiello et al., 2009). To clarify the role of TFEB in TNF-α treated microglia, we studied and found that a time-dependent TFEB translocation from cytosol to nuclear fraction in response to TNF-α compared with untreated BV2 cells (Figure 5E). Moreover, the lysosome proteins LAMP1 and LAMP2 expression also increased after TNF-α treatment (Figures 5F,G).

### AKT Signaling Is Involved in TNF-α Induced Autophagy Flux Disruption in Microglia

**FIGURE 6:**
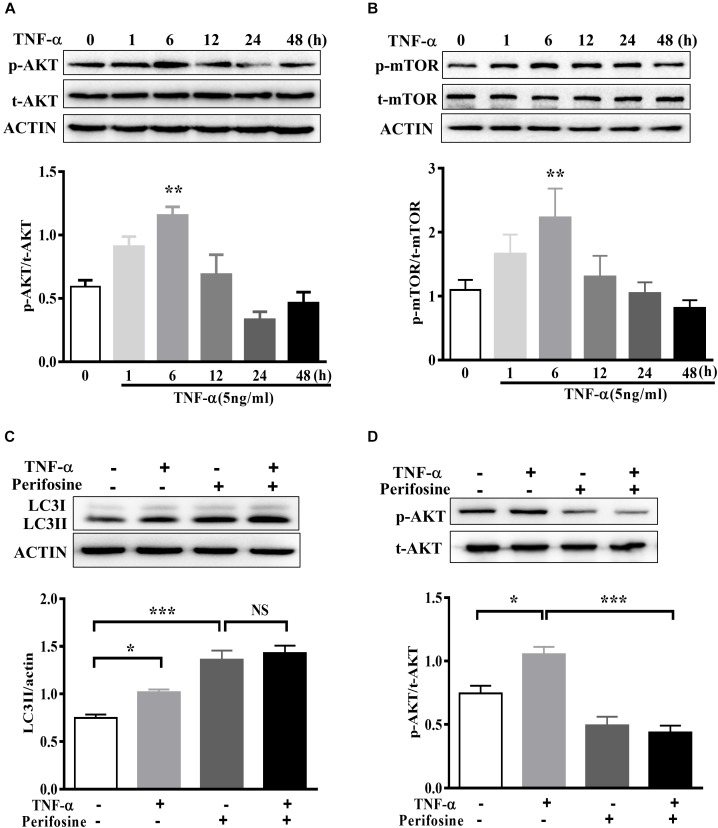
AKT signaling mediates TNF-α-induced autophagy inhibition. **(A,B)** Time-dependent effect of TNF-α on AKT and mTOR signaling. BV2 cells were treated with 5 ng/ml TNF-α for the indicating time. The changes of p-AKT, AKT, p-mTOR, and mTOR proteins level were analyzed by western blotting, *n* = 4. **(C,D)** Effect of TNF-α on LC3II protein level in the presence of AKT inhibitor perifosine (100 nM). *N* = 5. Actin served as loading controls. ^∗^*P* < 0.05, ^∗∗^*P* < 0.01, and ^∗∗∗^*P* < 0.001 vs. controls. One way ANOVA followed by Tukey analysis. NS, not significant.

The signaling mechanism that underlies TNF-α induced autophagy flux disruption was also examined. We observed a rapid activation of AKT in BV2 cells following 5 ng/ml TNF-α treatment, as AKT phosphorylation (Ser473) increased and reached its peak at 6 h after treatment (Figure 6A). This was accompanied by a consistent elevation in the phosphorylation of mTOR, the master regulator of autophagy and downstream target of AKT (Figure 6B). As expected, treatment with the AKT-specific inhibitor perifosine (100 nM) caused a LC3II increase, correlated with a reduction in Akt phosphorylation in BV2 cells. Of note, TNF-α did not further enhance the LC3II protein level in the presence of perifosine compared with perifosine treatment group (Figures 6C,D). This implies that the AKT/mTOR signaling pathway was involved in the autophagy regulation in TNF-α-stimulated microglia.

### TNF-α-Challenged Microglia Conditioned Medium Produced Toxicity to Co-cultured Neuronal Cells

**FIGURE 7:**
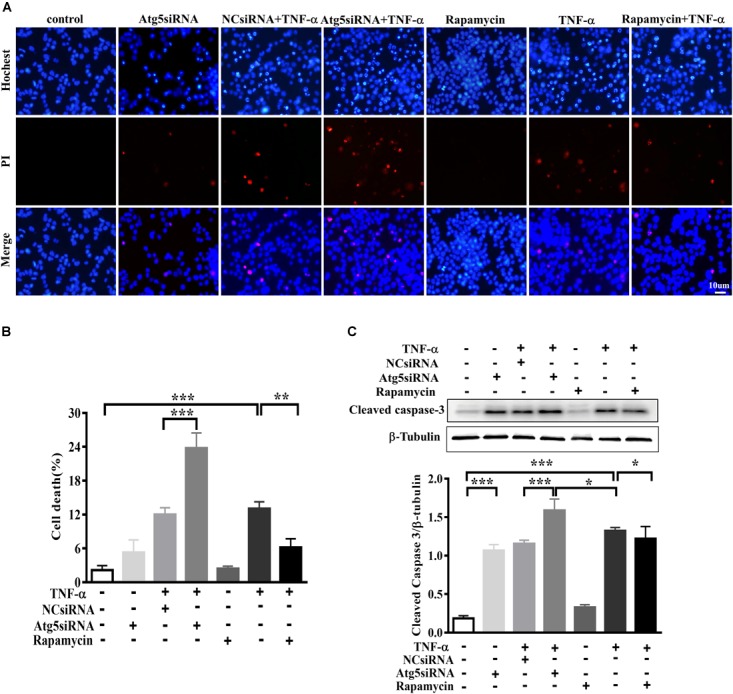
Effect of TNF-α-challenged microglia conditioned medium on dopaminergic cell damage. **(A–C)** MES23.5 cells were cultured for 24 h with different microglia CM as described in materials and methods. After that, MES23.5 cells were subjected to Hoechst/PI staining or western blot analysis of cleaved caspase-3 protein levels. The results were independently repeated three times. Scale bar, 10 μm.^∗^*P* < 0.05, ^∗∗^*P* < 0.01, and ^∗∗∗^*P* < 0.001 vs. controls. One way ANOVA followed by Tukey analysis.

In order to evaluate the effect of microglia polarization on neurons around, we established a system in which the cell-free culture supernatant from TNF-α or vehicle challenged BV2 cells (defined as a microglia conditioned medium) was harvested and then transferred into MES23.5 cells, a dopaminergic cell line. To assess the CM-associated impact and avoid the carryover from microglia treatment, BV2 cells were challenged with TNF-α for 24 h, washed, and cultured with fresh medium for another 24 h. After that, the microglia culture supernatant was collected as CM and applied in subsequent experimentation. To determine the influence of microglia autophagy regulation on neurons survival, BV2 cells were transfected with Atg5 siRNA or pretreated with rapamycin before exposure to TNF-α. The hochest and PI staining showed that treatment with TNF-α challenged CM for 24 h resulted in a 13.1 ± 1.2% cell death in MES23.5 cells compared to control CM group (2.2 ± 0.8%), implying TNF-α challenged CM produced toxicity to neuronal cells. This neurotoxicity was enhanced by Atg5 knockdown but attenuated when BV2 cells pretreatment with rapamycin (Figures 7A,B). The results were confirmed with the caspase-3 assay. Western blotting showed that treatment with TNF-α challenged CM caused an increase of cleaved caspase-3 level in MES23.5 cells. Transfection with Atg5 siRNA in BV2 microglia led to a further increase in cleaved caspase-3 compared to control siRNA. In contrast, microglia pretreatment with rapamycin attenuated the cleaved caspase-3 increase (Figure 7C). These results indicate that modulation of microglia autophagy could have an impact on the neuronal survival probably by affecting microglia polarization and associated neuroinflammation.

## Discussion

**FIGURE 8:**
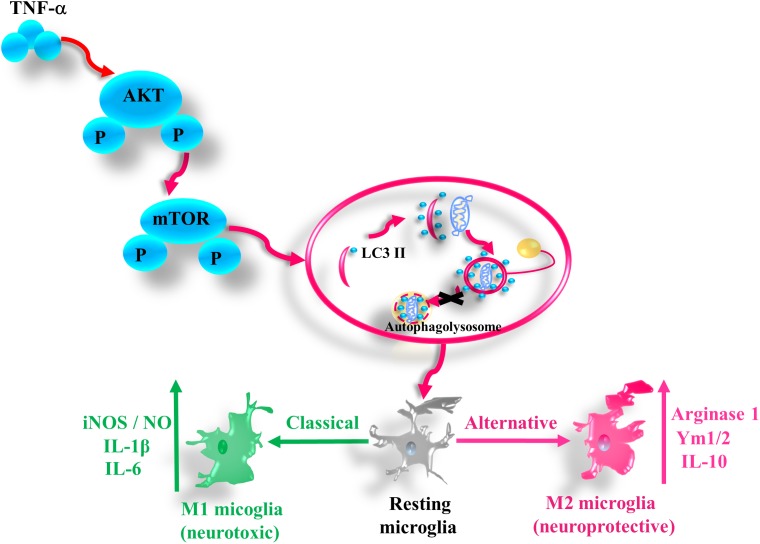
A simplified summary of TNF-α induced autophagy flux inhibition and subsequent inflammation through AKT/mTOR signaling in microglia. TNF-α triggers the phosphorylation of AKT and mTOR, which plays a role in the autophagy flux disruption and causes a shift toward M1 phenotype in microglia. As a result, the microglia expression of the M1 markers such as iNOS/NO, IL-6, and IL-1β increases, while that of M2 genes including arginase, Ym1/2, and IL-10 decreases.

In this study we provide the evidence that pro-inflammatory cytokine TNF-α inhibits autophagy flux and drives microglia shift toward M1 phenotype via activating AKT/mTOR signaling. Our findings demonstrate that autophagy enhancement could guide microglia activation toward M2 status and attenuate the neurotoxicity of TNF-α challenged microglia CM, while microglia autophagy inhibition produces the opposite effects (as summarized in Figure 8).

Neuroinflammation and autophagy dysregulation closely linked with neurodegeneration. Recent studies identify a complex interaction between these two processes. Here we clearly show that TNF-α inhibits microglia autophagy flux, and reveal a role of autophagy flux inhibition in M1 microglia polarization and thereby the neurotoxicity. This is consistent with recent reports that autophagy may regulate LPS-stimulated inflammation in microglia and its associated neurotoxicity (Bussi et al., 2017; He et al., 2018). These lines of evidence highlight a possibility that autophagy serves as the common machinery in regulating microglia-mediated inflammation in response to a multitude of inflammatory stimuli. Yet, another study demonstrated that LPS injection induced autophagy activation in rat hippocampus (Pintado et al., 2017). Autophagy is a catabolic process that can be activated during starvation, stress and nutrient deprivation by different signaling pathways. It is conceivable that microglia autophagy could be altered by diverse conditions. Also, the isolation and culture conditions could yield an impact on microglia *in vitro*. Thus, the *in vivo* study, in combination with microglia-specific genetic manipulation, may further our understanding of the relationship between inflammation and autophagy.

We previously reported that TNF-α can disrupt the autophagic flux and result in α-synuclein accumulation in PC12 cells and midbrain neurons (Wang et al., 2015). This and the present study consistently show TNF-α causes autophagy dysfunction in both neuron and microglia, which may produce a positive loop between microglia and neurons and thus lead to neurodegeneration. However, we did not observe any change in TFEB or LAMP1 expression in TNF-α-treated neurons (Wang et al., 2015). By contrast, in this study we detected TFEB nuclear translocation induced by TNF-α in microglia, correlating with the increases of LAMP1 and LAMP2 expression. This may act as a compensatory mechanism to autophagy flux disruption during TNF-α stimulation. This discrepancy may be attributed to the higher activity of lysosomal biogenesis in microglia. The TNF-α induced autophagy flux inhibition seemed to be dependent, at least in part, on AKT activation. An early activation of AKT, accompanied by mTOR activation, was observed in microglia after TNF-α stimulation. In the presence of the AKT inhibitor, TNF-α failed to alter the autophagic marker LC3II. Therefore, activation of AKT/mTOR signaling may contribute to TNF-α-induced autophagy inhibition in microglia. A recent study also identifies the role of AKT activation in regulating autophagy during the late stage (Matsuda-Lennikov et al., 2014). Phafin2 (EAPF or PLEKHF2), a lysosomal protein interacts with and recruits cytosolic Akt to the lysosome, and thus affects the autophagy-lysosome pathway.

The identification of different phenotypes is a breakthrough in microglia biology. M1 or M2 polarization reflects different microglia functions. Generally, M1 is pro-inflammatory and neurotoxic while M2 is anti-inflammatory and neuroprotective (Moehle and West, 2015). This opens up a novel avenue for the treatment of neuroinflammation related disorders. Notably, M1 or M2 markers are not exclusively expressed in microglia. Their phenotypic changes during neurodegeneration may be driven by extracellular and intracellular factors. The extracellular triggers were extensively reported, such as beta-amyloid and LPS (Qin et al., 2007; Hickman et al., 2008). Only a few molecules including AMPK, hydrogen sulfide, and A1 adenosine receptor, have been reported to modulate microglia polarization (Tsutsui et al., 2004; Zhou et al., 2014; Xu et al., 2015). Hence, the intracellular machinery warrants intensive study. Our study reveals autophagy as an endogenous “brake” on microglia phenotype shift toward M1 polarization. Atg5 (an autophagy-related protein) knockdown was sufficient to trigger M1 microglia activation in the absence of inflammatory stimulation. In TNF-α-challenged microglia, autophagic flux was impaired, associated with the increase of M1 marker (iNOS/NO, IL-1β, and IL-6) and the decrease in M2 marker (Arginase1, Ym1/2, and IL-10) expression. Upregulation of autophagy with serum deprivation or with pharmacologic activators (rapamycin and resveratrol) promoted microglia polarization toward M2 phenotype while inhibition of autophagy with 3-MA or Atg5 siRNA consistently aggravated the M1 polarization induced by TNF-α. These findings suggest that autophagy acts as an endogenous “brake” on microglia shift toward M1 in normal situation. In disease conditions, microglia autophagy deficiency may sensitize the cells to simulation and boost neuroinflammation.

Persistent microglia activation toward M1 phenotype could have physiologic relevance in brain regions with high microglia density such as SN (Pintado et al., 2011; Leal et al., 2013). This may contribute to dopaminergic neurodegeneration in PD. In support of this, here we found TNF-α-challenged microglia CM produced toxicity to the cocultured MES23.5 cells. The neurotoxicity was boosted if inhibiting microglia autophagy, but alleviated when microglia autophagy was activated. This CM-derived toxicity may derive from an accumulation of neurotoxic and pro-inflammatory molecules in the challenged CM. Consistently, the cytokines assay (Figures 2, 3) demonstrated a role of autophagy in modulating cytokines generation in microglia. Sure, other untested factors may also contribute to the toxicity.

In sum, our study demonstrates that TNF-α inhibits autophagy flux in microglia through activating AKT/mTOR signaling pathway, and identifies that autophagy serves as a negative regulator of microglia polarization in both normal and inflamed conditions. This study also highlights the potential of autophagy inducers in the treatment of neuroinflammation-associated degeneration.

## Author Contributions

C-FL and L-FH designed this study. M-mJ, FW, and W-wL carried out all the experiments. CG, DQ, ZZ, C-JM, and Y-PY revised the manuscript. L-FH and M-mJ contributed to writing the manuscript. All authors read and approved the final version of the manuscript.

## Conflict of Interest Statement

The authors declare that the research was conducted in the absence of any commercial or financial relationships that could be construed as a potential conflict of interest.

## References

[B1] Bussi C. Peralta Ramos J. M. Arroyo D. S. Gaviglio E. A. Gallea J. I. Wang J. M. (2017). Autophagy down regulates pro-inflammatory mediators in BV2 microglial cells and rescues both LPS and alpha-synuclein induced neuronal cell death. *Sci. Rep.* 7:43153. 10.1038/srep43153 28256519 PMC5335665

[B2] Du C. Jin M. Hong Y. Li Q. Wang X. H. Xu J. M. (2014). Downregulation of cystathionine beta-synthase/hydrogen sulfide contributes to rotenone-induced microglia polarization toward M1 type. *Biochem. Biophys. Res. Commun.* 451 239–245. 10.1016/j.bbrc.2014.07.107 25086357

[B3] Fujita K. Maeda D. Xiao Q. Srinivasula S. M. (2011). Nrf2-mediated induction of p62 controls Toll-like receptor-4-driven aggresome-like induced structure formation and autophagic degradation. *Proc. Natl. Acad. Sci. U.S.A.* 108 1427–1432. 10.1073/pnas.1014156108 21220332 PMC3029726

[B4] Glass C. K. Saijo K. Winner B. Marchetto M. C. Gage F. H. (2010). Mechanisms underlying inflammation in neurodegeneration. *Cell* 140 918–934. 10.1016/j.cell.2010.02.016 20303880 PMC2873093

[B5] He Y. She H. Zhang T. Xu H. Cheng L. Yepes M. (2018). p38 MAPK inhibits autophagy and promotes microglial inflammatory responses by phosphorylating ULK1. *J. Cell Biol.* 217 315–328. 10.1083/jcb.201701049 29196462 PMC5748971

[B6] Hickman S. E. Allison E. K. El Khoury J. (2008). Microglial dysfunction and defective beta-amyloid clearance pathways in aging Alzheimer’s disease mice. *J. Neurosci.* 28 8354–8360. 10.1523/JNEUROSCI.0616-08.2008 18701698 PMC2597474

[B7] Hu X. Li P. Guo Y. Wang H. Leak R. K. Chen S. (2012). Microglia/macrophage polarization dynamics reveal novel mechanism of injury expansion after focal cerebral ischemia. *Stroke* 43 3063–3070. 10.1161/STROKEAHA.112.659656 22933588

[B8] Hunot S. Hirsch E. C. (2003). Neuroinflammatory processes in Parkinson’s disease. *Ann. Neurol.* 53(Suppl. 3), S49–S58. 10.1002/ana.10481 12666098

[B9] Kigerl K. A. Gensel J. C. Ankeny D. P. Alexander J. K. Donnelly D. J. Popovich P. G. (2009). Identification of two distinct macrophage subsets with divergent effects causing either neurotoxicity or regeneration in the injured mouse spinal cord. *J. Neurosci.* 29 13435–13444. 10.1523/JNEUROSCI.3257-09.2009 19864556 PMC2788152

[B10] Kim W. G. Mohney R. P. Wilson B. Jeohn G. H. Liu B. Hong J. S. (2000). Regional difference in susceptibility to lipopolysaccharide-induced neurotoxicity in the rat brain: role of microglia. *J. Neurosci.* 20 6309–6316. 10.1523/JNEUROSCI.20-16-06309.2000 10934283 PMC6772569

[B11] Leal M. C. Casabona J. C. Puntel M. Pitossi F. J. (2013). Interleukin-1beta and tumor necrosis factor-alpha: reliable targets for protective therapies in Parkinson’s disease? *Front. Cell Neurosci.* 7:53 10.3389/fncel.2013.00053 PMC3638129 23641196

[B12] Mantovani A. Biswas S. K. Galdiero M. R. Sica A. Locati M. (2013). Macrophage plasticity and polarization in tissue repair and remodelling. *J. Pathol.* 229 176–185. 10.1002/path.4133 23096265

[B13] Matsuda-Lennikov M. Suizu F. Hirata N. Hashimoto M. Kimura K. Nagamine T. (2014). Lysosomal interaction of Akt with Phafin2: a critical step in the induction of autophagy. *PLoS One* 9:e79795. 10.1371/journal.pone.0079795 24416124 PMC3885392

[B14] Mccoy M. K. Ruhn K. A. Blesch A. Tansey M. G. (2011). TNF: a key neuroinflammatory mediator of neurotoxicity and neurodegeneration in models of Parkinson’s disease. *Adv. Exp. Med. Biol.* 691 539–540. 10.1007/978-1-4419-6612-4_56 21153359 PMC12289335

[B15] Menza M. Dobkin R. D. Marin H. Mark M. H. Gara M. Bienfait K. (2010). The role of inflammatory cytokines in cognition and other non-motor symptoms of Parkinson’s disease. *Psychosomatics* 51 474–479. 10.1176/appi.psy.51.6.474 21051678 PMC2987579

[B16] Mizushima N. (2007). Autophagy: process and function. *Genes Dev.* 21 2861–2873. 10.1101/gad.1599207 18006683

[B17] Mizushima N. Yoshimori T. Levine B. (2010). Methods in mammalian autophagy research. *Cell* 140 313–326. 10.1016/j.cell.2010.01.028 20144757 PMC2852113

[B18] Moehle M. S. West A. B. (2015). M1 and M2 immune activation in Parkinson’s disease: foe and ally? *Neuroscience* 302 59–73. 10.1016/j.neuroscience.2014.11.018 25463515 PMC4442748

[B19] Orihuela R. Mcpherson C. A. Harry G. J. (2016). Microglial M1/M2 polarization and metabolic states. *Br. J. Pharmacol.* 173 649–665. 10.1111/bph.13139 25800044 PMC4742299

[B20] Pintado C. Macias S. Dominguez-Martin H. Castano A. Ruano D. (2017). Neuroinflammation alters cellular proteostasis by producing endoplasmic reticulum stress, autophagy activation and disrupting ERAD activation. *Sci. Rep.* 7:8100. 10.1038/s41598-017-08722-3 28808322 PMC5556015

[B21] Pintado C. Revilla E. Vizuete M. L. Jimenez S. Garcia-Cuervo L. Vitorica J. (2011). Regional difference in inflammatory response to LPS-injection in the brain: role of microglia cell density. *J. Neuroimmunol.* 238 44–51. 10.1016/j.jneuroim.2011.06.017 21803430

[B22] Plaza-Zabala A. Sierra-Torre V. Sierra A. (2017). Autophagy and microglia: novel partners in neurodegeneration and aging. *Int. J. Mol. Sci.* 18:E598. 10.3390/ijms18030598 28282924 PMC5372614

[B23] Qin L. Wu X. Block M. L. Liu Y. Breese G. R. Hong J. S. (2007). Systemic LPS causes chronic neuroinflammation and progressive neurodegeneration. *Glia* 55 453–462. 10.1002/glia.20467 17203472 PMC2871685

[B24] Saitoh T. Fujita N. Jang M. H. Uematsu S. Yang B. G. Satoh T. (2008). Loss of the autophagy protein Atg16L1 enhances endotoxin-induced IL-1beta production. *Nature* 456 264–268. 10.1038/nature07383 18849965

[B25] Sardiello M. Palmieri M. Di Ronza A. Medina D. L. Valenza M. Gennarino V. A. (2009). A gene network regulating lysosomal biogenesis and function. *Science* 325 473–477. 10.1126/science.1174447 19556463

[B26] Tsutsui S. Schnermann J. Noorbakhsh F. Henry S. Yong V. W. Winston B. W. (2004). A1 adenosine receptor upregulation and activation attenuates neuroinflammation and demyelination in a model of multiple sclerosis. *J. Neurosci.* 24 1521–1529. 10.1523/JNEUROSCI.4271-03.2004 14960625 PMC6730323

[B27] Vivekanantham S. Shah S. Dewji R. Dewji A. Khatri C. Ologunde R. (2015). Neuroinflammation in Parkinson’s disease: role in neurodegeneration and tissue repair. *Int. J. Neurosci.* 125 717–725. 10.3109/00207454.2014.982795 25364880

[B28] Wang M. X. Cheng X. Y. Jin M. Cao Y. L. Yang Y. P. Wang J. D. (2015). TNF compromises lysosome acidification and reduces alpha-synuclein degradation via autophagy in dopaminergic cells. *Exp. Neurol.* 271 112–121. 10.1016/j.expneurol.2015.05.008 26001614

[B29] Wang Y. Li Y. Li H. Song H. Zhai N. Lou L. (2017). Brucella Dysregulates Monocytes and Inhibits Macrophage Polarization through LC3-Dependent Autophagy. *Front. Immunol.* 8:691. 10.3389/fimmu.2017.00691 28659924 PMC5467008

[B30] Wyss-Coray T. Rogers J. (2012). Inflammation in Alzheimer disease-a brief review of the basic science and clinical literature. *Cold Spring Harb. Perspect. Med.* 2:a006346. 10.1101/cshperspect.a006346 22315714 PMC3253025

[B31] Xu Y. Xu Y. Wang Y. Wang Y. He L. Jiang Z. (2015). Telmisartan prevention of LPS-induced microglia activation involves M2 microglia polarization via CaMKKbeta-dependent AMPK activation. *Brain Behav. Immun.* 50 298–313. 10.1016/j.bbi.2015.07.015 26188187

[B32] Zhou X. Cao Y. Ao G. Hu L. Liu H. Wu J. (2014). CaMKKbeta-dependent activation of AMP-activated protein kinase is critical to suppressive effects of hydrogen sulfide on neuroinflammation. *Antioxid. Redox. Signal.* 21 1741–1758. 10.1089/ars.2013.5587 24624937 PMC5695757

